# From Doctor to Nurse Triage in the Danish Out-of-Hours Primary Care Service: Simulated Effects on Costs

**DOI:** 10.1155/2013/987834

**Published:** 2013-09-30

**Authors:** Grete Moth, Linda Huibers, Peter Vedsted

**Affiliations:** ^1^The Research Unit for General Practice, Aarhus University, Bartholins Allé 2, 8000 Aarhus C, Denmark; ^2^Scientific Institute for Quality of Healthcare, Radboud University Nijmegen Medical Centre, Geert Grooteplein 9, 6500 HB Nijmegen, The Netherlands

## Abstract

*Introduction*. General practitioners (GP) answer calls to the Danish out-of-hours primary care service (OOH) in Denmark, and this is a subject of discussions about quality and cost-effectiveness. The aim of this study was to estimate changes in fee costs if nurses substituted the GPs. *Methods*. We applied experiences from The Netherlands on nurse performance in the OOH triage concerning the number of calls per hour. Using the 2011 number of calls in one region, we examined three hypothetical scenarios with nurse triage and calculated the differences in fee costs. *Results*. A new organisation with 97 employed nurses would be needed. Fewer telephone consultations may result in an increase of face-to-face contacts, resulting in an increase of 23.6% in costs fees. Under optimal circumstances (e.g., a lower demand for OOH services, a high telephone termination rate, and unchanged GP fees) the costs could be reduced by 26.2% though excluding administrative costs of a new organisation. *Conclusion*. Substituting GPs with nurses in OOH primary care may increase the cost in fees compared to a model with only GPs. Further research is needed involving more influencing factors, such as costs due to nurse training and running the organisation.

## 1. Introduction

Many of the European out-of-hours (OOH) services are to some degree primary care based [1, 2], as the populations' need for medical advice is often best met using a family medicine approach, except in emergency cases. In Denmark, a reform of the OOH primary care services in 1992 resulted in a considerable substitution of home visits by general practitioner (GP) telephone consultations and local GP clinic consultations [3, 4]. The OOH primary care is GP driven, based on a public scheme where GPs share the responsibility of providing care each weekday from 4 p.m. to 8  a.m. and in weekends and on bank holidays [4]. Remuneration is based fully on a fee-for-service that varies by type of contact, receiving the highest fee for home visits and a higher fee for ending contacts on the phone as compared with referral to a subsequent face-to-face contact.

Following the Danish reform in 1992, OOH primary care in, for example, The Netherlands and the United Kingdom was reformed into large-scale GP cooperatives inspired by the Danish solution [5, 6]. Danish GPs perform all clinical tasks themselves, whereas, in The Netherlands, nurses and nurse assistants answer patient calls and perform telephone triage. Supervised by a GP, the nurses/nurse assistants decide whether the patients are to be given a telephone advice or are to be examined by a GP at a subsequent clinic consultation or home visit. They are qualified and certified through training in communicative and practical triage skills and have access to a guideline-based computer-assisted decision tool to support the triage [7, 8]. Concerning the level of urgency of calls in the two countries a study of patient safety in The Netherlands showed that 59.9% of calls were noneurgent (routine, no time pressure), and similarly in Denmark a survey on reasons for encounter in the OOH primary care showed that 59.1% of calls were considered noneurgent (not severe, not ill) [9, 10].

In recent years, the organisation of the OOH with all tasks being managed by the GPs has been a political issue in Denmark. In particular, substitution of the GPs with fully trained nurses in the telephone triage has been discussed. It is expected to result in a cut-down of expenses, as nurse fees are lower than fees of GPs. However, as there have been no studies on this we need better knowledge on this specific argument.

We aimed to examine whether introducing nurse-based triage in the Danish OOH primary care would carry a decrease in costs based on the Dutch experiences.

## 2. Methods

### 2.1. Design

**Figure 1:**
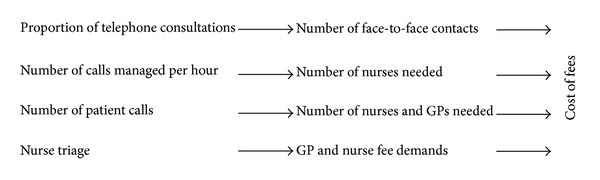
A schematic illustration of the factors influencing the cost of fees.

We constructed three scenarios of nurse substitution in Danish OOH primary care taking into account various factors influencing the patient flows and the effects on costs (Figure 1). As the organisation for OOH primary care is quite similar in Denmark and The Netherlands, we used input from the Dutch services to simulate the effects.

### 2.2. Basis for the Simulation

As a basis for comparing the hypothetical scenarios with the existing situation we used information on the number of patient contacts and distribution of type of contacts in 2011 in the existing OOH primary care organisation in the Central Denmark Region. This information was based on data from the Health Insurance Registry [11], being available on the website of Statistics Denmark, the central authority on Danish statistics [12]. This region has a population of 1.25 million, which is approximately one-fifth of the Danish population [13].

### 2.3. Estimates Needed for the Simulations

Two estimates were crucial for the calculations: the proportion of terminated telephone consultations and the number of managed calls per hour. The percentage of contacts ended by telephone in case of nurse-based triage was based on the performance of Dutch triage nurses in OOH primary care. According to the annual benchmark of the Dutch association of general practitioners cooperatives (the VHN) the triage nurses terminate 41% of calls on the phone [14]. A Dutch study showed that the average duration of a call was five minutes, implying a rate of 12 calls per hour [13]. However, this estimate does not take into account breaks, time for documentation, conference with the supervising GP, or waiting time in periods with few calls. Therefore, we used the estimate of six calls per hour based on information from core persons in the Dutch OOH primary care and research (source: the authors of a report about the workload at a GP cooperative) [15]. 

### 2.4. Scenarios

For the simulation, we set up three scenarios.

**Scenario**

The number of calls to the Danish OOH primary care was unchanged from the existing model. Nurses could terminate 18 percent less of the calls compared with the GPs (41% versus 59%), thus implying a rise of the number of consultations and home visits (Table 1).

**Scenario**

The introduction of nurse triage would result in fewer calls to OOH primary care (i.e., 75% of the present number), based on the theory that parts of the calls were related to the direct telephone access to a GP and not to an acute need for help.

**Scenario**

Like Scenario 2, but here nurses were able to terminate the same proportion of telephone calls as the GPs.

### 2.5. Costs

The costs of the present organisation were based on data from the Health Insurance Registry [11] on GP remuneration [16]. In the simulated scenarios, we assumed that GPs doing clinic consultations and home visits were paid a fee-for-service as in the existing organisation. These are results of dividing the total costs [16] with the number of contacts for each type of contact [12] and multiplicating the number of contacts in the scenarios.

For calculating the fees for nurses in the simulated models, we used the fees for the nurses doing telephone triage at the emergency department (ED), as the characteristics of these tasks are quite similar. We used the fees and pay supplements of the official agreement for nurse salaries including the agreed pension and holiday payment, that is, fixed salaries per hour and not fee-for-service as for the GPs, as fee-for-service for nurses is not a realistic option. Danish nurses are entitled to additional days off due to working after hours, according to the official agreement. This was added in the simulation (i.e., four hours per eight hours on Sundays and one hour per eight hours on all other OOH shifts). Moreover, we estimated absence due to days off work, holidays, illness, and so forth to eight weeks per nurse per year.

The number of nurses needed was calculated as follows: in 2011, the mean number of calls per eight-hour shift to the OOH primary care in the Central Denmark Region was 755 (94 calls per hour). Considering the estimated number of calls manageable by nurses, a mean number of 16 nurses would be needed per hour. As the number of working hours per shift is eight hours, this resulted in a need for a total number of 105,830 nurse working hours annually. Furthermore, the days off due to OOH work and the estimated absence resulted in a total of 97 nurses to cover one year. In simulations 2 and 3, we decreased the number of calls to OOH care, resulting in a number of nurses needed to be 76.

Moreover, we followed the Dutch organisation with a GP supervisor for the triage nurses for a fixed salary. Therefore, we counted the number of shifts in the study period and applied one GP for each shift in the scenarios. As GPs currently do not receive fixed salaries in OOH care, we based the estimation of these costs on the centrally agreed fees per hour for doctors acting as consultants. 

The calculated costs comprised the fees of GPs and nurses only and did not include additional costs due to organisation, transportation, or administration of the nurse schemes, training, maternity leave, and continuous education. We used the exchange rate of 13.4 Euros per 100 DKK (February 2013).

## 3. Results 

### 3.1. Existing Situation

In 2011, 634,978 patient calls to the OOH primary care were registered in Central Denmark Region. Table 1 shows that of these contacts, 375,403 (59%) were terminated as telephone consultations with no further contact. Accordingly, the remaining 259,575 (41%) had a consultation (178,851 (28%)) or a home visit (80,724 (31%)). The cost with regard to GP fees was € 12,899,196 in total for the existing situation.

### 3.2. Scenarios

Table 1 shows the fee costs for the different scenarios and the existing OOH. In Scenario 1 (i.e., less contacts terminated on the telephone (59% to 41%) and equal number of patient calls), the total costs were € 15,939,834, which was an increase in costs of 23.6%. In Scenario 2 (i.e., lower demand (calls) and the share of terminated telephone consultations as in the existing model (model 1)), the total costs were € 12,330,202, which was a 4.4% decrease in costs. In Scenario 3 (i.e., less demand and same share of terminated telephone consultations as in the existing model), the total costs were € 9,522,563 which resulted in a 26.2% decrease in costs.

## 4. Discussion

### 4.1. Main Findings

We found that, in a scenario with nurses substituting GPs in the OOH primary care telephone triage with the same number of contacts to the OOH primary care service and an 18 percent decrease in contacts terminated on the phone, the costs would increase by 23.6% (approximately three mill Euros) compared with the existing situation. Sensitivity analyses, using a number of assumptions, showed that the most optimistic scenario (i.e., a 25% fall in calls and triage nurses being as efficient in terminating telephone calls as the GPs) would result in a 26.2% costs reduction. In our cost calculations, we have been conservative as unknown factors, such as costs due to training the nurses, running the organisation, and specific calculations of the number of supervising GPs, were not included and may add considerably to the final costs.

### 4.2. Discussion of Methods and Results

Setting up simulated scenarios like the ones presented here implies incorporating assumptions and risking simplification of things. Additional simulated models with varying patient flows, numbers of nurses needed, and nurse fees could have been integrated in the comparison.

The presented scenarios are based on existing data from a large OOH primary care service and on knowledge of the performance of triage nurses in Dutch OOH primary care, which in many ways is comparable to Denmark. Study results on patients' reasons for calling the OOH primary care are sparse. An international study comparing reasons for encounter in the OOH primary care in a range of European countries found that Denmark and The Netherlands were quite comparable in this respect [1]. Denmark has a higher consumption rate of 535 OOH services per 1,000 inhabitants per year compared with 238 contacts in The Netherlands [17, 18]. Therefore, we incorporated less demand from citizens in one of the sensitivity analyses, thus taking qualified estimated variations into consideration in the attempt to make realistic scenarios. Likewise, we allowed for a more effective nurse triage by estimating a percentage of terminated telephone consultations equal to the GPs. We might also have added a scenario with an increase in calls due to nurses answering the call. However, as the Danish consumption pattern within OOH primary care already at present is high, we considered such a scenario as unrealistic.

The number of nurses needed may be over- or underestimated. We could have chosen to use the performance of Danish ED-triage nurses as a basis for comparison instead of using Dutch OOH triage staff. However, as patient calls to the ED-nurses are likely to differ significantly from the calls to OOH primary care we considered this as a less suitable option. As the number of calls per 1,000 Danish citizens is twice as big as the number of calls in The Netherlands [17], the amount of severe and complex calls may be smaller. This may imply that Danish triage nurses can perform more effectively, exceeding six calls per hour and terminating more calls on the telephone. On the other hand, if the number of calls decreases because GPs are no longer directly available on the phone, these calls may contain a larger share of more serious problems needing a GP contact. If this is the case, nurses may spend more time on the calls and/or to send more patients to a subsequent face-to-face contact, resulting in higher expenses.

The fees used in the simulated scenario calculation are based on the minimum level of fees for nurses doing triage to the ED. In reality, many of the nurses can be expected to have additional specialist courses and longer working hours, which will prompt demands for higher fees. Furthermore, we have not included extra costs due to training of nurses, license of a computerised support system, and administration/management of a new organisation, including a head of the department. Such costs will be additionally compared with the existing situation, as these organisational tasks currently are paid for by all GPs in the region at an amount of 269 Euros per year and managed by an OOH board of GPs. As the GPs are paid a fee for service in the existing model, no extra costs are related to sickness or holidays of GPs because if absent another GP takes over with no additional costs involved. GP transportation costs for home visits are not included in the simulated models. If, as assumed, the number of home visits increases this will add to the total costs.

The Danish OOH primary care organisations are remarkably larger than the Dutch, covering a much larger service area. In the scenarios, we included the fees for only one supervising GP. However, as the number of nurses needed in the call centre can be expected to be much higher than in the Dutch GP cooperatives, one supervising GP is probably not enough in periods of high call demands. This is the case in which the fees for supervising GPs can be expected to increase from the present 781,000 Euros in the scenarios.

We have not been able to simulate possible effects on service and health induced by these different scenarios. However, it is possible that citizens might experience changes in service and medical advice and that there will be a changed demand for, for example, emergency calls.

Nurses answer calls and perform triage in general practice in daytime. However, there is a fundamental difference, as patients calling during daytime are listed with the practice, meaning that the staff has access to the patient records. It is likely that decision support systems equivalent to those known from other countries will make the nurses more qualified to perform the triage [19–21]. Yet, the Dutch nurses, whose performance is used in this comparison, already make use of an electronic decision support system which has not raised the level of telephone consultations to the Danish GP level. 

## 5. Conclusion

This study suggests that the cost-economic consequences of substituting GPs with nurses in the Danish OOH service are dependent on a range of factors. Under certain optimal circumstances (e.g., a lower demand for OOH services due to nurses answering the telephone instead of GPs and a high telephone termination rate) nurse triage may result in a decrease in costs. However, nurse triage can be expected to lead more face-to-face contacts resulting in higher GP fees. Additionally, taking costs to build and run a new organisation into consideration should be included in future calculations of the total expenses of changing the organisation to introduce nurse triage.

## Figures and Tables

**Table 1:** The existing situation and four simulated models of patient flows in the regional out-of-hours service taking care of 1.25 mill inhabitants presented in number of contacts and costs during a year. Based on figures from 2011.

	Telephone consultations^2^	Supervising GPs	Clinic consultations	Home visits	Total costs (€)
Existing situation: (634,978 patient calls^1^)					
Present flow					
Share	59%		28%	13%	
Numbers	375,403		178,851	80,724	
Present costs					
Costs (€)	5,016,086		4,792,761	3,090,349	12,899,196

Scenario 1: (634,978 patient calls^1^ = 100% of calls)					
Simulated flow					
Share	41%		40%	19%	
Numbers	260,341		253,991	120,646	
Simulated costs					
Costs (€)	3,742,825	772,006	6,806,332	4,618,671	15,939,834

Scenario 2: (476,234 patient calls = 75% of calls)					
Simulated flow					
Share	41%		40%	19%	
Numbers	195,256		190,494	90,484	
Simulated costs					
Costs (€)	2,807,119	772,006	5,104,754	3,646,323	12,330,202

Scenario 3: (476,234 patient calls = 75% of calls)					
Simulated flow					
Share	59%		28%	13%	
Numbers	280,978		133,346	61,910	
Simulated costs					
Costs (€)	2,807,119	772,006	3,573,328	2,370,110	9,522,563

^1^The number of patients calling the out-of-hours service in 2011 used in the scenarios.

^2^Contacts terminated on the telephone.
